# 

**Published:** 1890-11-08

**Authors:** 


WITH EXTRA NURSING SUPPLEMENT.

Per Annum, 6s. 6d.; post free.

Monthly Parts, 6d.; post free 8d.

Ditto per Annum, 8s., post free.

### THE HOSPITAL

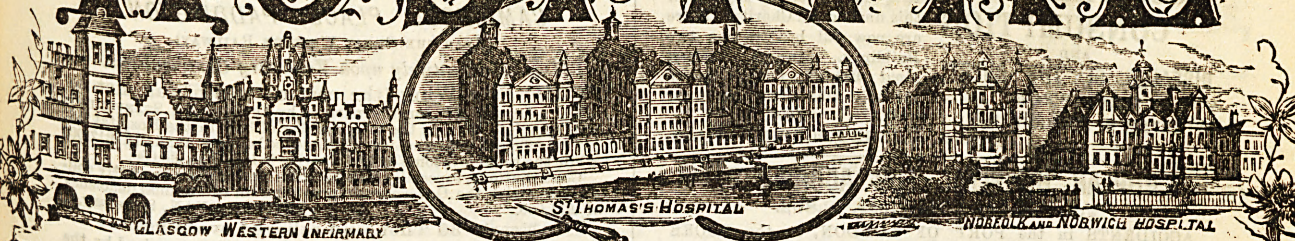

A WEEKLY JOURNAL OF

### Science, Medicine, Nursing and Philanthropy.

SATURDAY, NOVEMBER 8th, 1890.

**What not to Expect from Koch** - - - - - - 85
**Passing Topics.**—Fifteen per Cent. - - - - - 86
**The Doctor's Armchair.**—Can Science Resolve the Doubt? 87
**The Two Oldest English Medical Books** - - - 88
**Eyes and Noses** - - - - - - - - 89
**Working Women in Large Towns.**—XV.—Social, Moral, and Physical Aspects of the Subject - - - - 90
**Everybody's Page** - - - - - - - 91
**Notes and News** - - - - - - - 92
**The Story of the Insane from Year to Year** - - 93
**Annotations.**—Facts about Tubercle—Voluntary Patients in Lunatic Asylums—Starvation and Indigestion - - 94
**The Practitioner's Mirror and Retrospect:**—
The Pathological Society of London—A Case of Supposed Indigenous Leprosy - - - - - - 95
The London Post-Graduate Course at the Paddington Infirmary—Some Nerve Diseases—Pathology - - - 96
**Medical Schools**—Bristol - - - - - - 97
**The Hospitals of Bristol** - - - - - - 98
**The Student of Medicine:**—Students Past and Present—Men of the Future (with Portrait)—Notes from the Schools—Students' Medical Societies—Scholarships and Prizes—Pass Lists—Athletics - - - - - - - 100
**General Charities.**

### EXTRA SUPPLEMENT—THE NURSING MIRROR.

**En Passant.**—Sick Nurses—Asylum Attendants—English Nurses in Egypt—Taunton Victoria Institute—News from Rugby—Short Items - - - - - - - xxiii

**Lectures on Surgical Ward Work and Nursing.** By Alex. Miles, M.B.Edin., C.M., F.R.C.S.E.—Lecture II. Preventing Sepsis - - - - - - xxiv

**Presentations** - - - - - - - xxiv

**Words of Consolation.**—For the Aged - - - xxv

**Death in our Ranks** - - - - - - xxv

**Nurses on Their Travels.**—Notes from Australia - xxv

**Lectures** - - - - - - - - xxvi

**Everybody's Opinion.**—Males Nurses—The Tynemouth Infirmary—The "Special Pro's" Articles: A Protest, xxvi; Ladies on Committees—Norfolk and Norwich Hospitals—Cottage Nurses - - - - - xxvii

**Competitions—Notes and Queries** - - - xxvii

**Off Duty.**—An Eventful Night - - - - xxviii

**Amusements and Relaxation** - - - - xxviii

